# Influence of Hydrogen Electron Donor, Alkaline pH, and High Nitrate Concentrations on Microbial Denitrification: A Review

**DOI:** 10.3390/ijms20205163

**Published:** 2019-10-18

**Authors:** Pierre Albina, Nadège Durban, Alexandra Bertron, Achim Albrecht, Jean-Charles Robinet, Benjamin Erable

**Affiliations:** 1 Laboratoire Matériaux et Durabilité des Constructions, Université de Toulouse, UPS, INSA. 135, 7 avenue de Rangueil, 31077 Toulouse CEDEX 04, France; nadege.durban@gmail.com (N.D.); bertron@insa-toulouse.fr (A.B.); 2 Laboratoire de Génie Chimique, Université de Toulouse, CNRS, INPT, UPS, 31030 Toulouse, France; benjamin.erable@ensiacet.fr; 3 Andra (Agence nationale pour la gestion des déchets radioactifs), 92298 Châtenay-Malabry, France; achim.albrecht@andra.fr (A.A.); jean-charles.robinet@andra.fr (J.-C.R.)

**Keywords:** hydrogenotrophic denitrification, high pH, high nitrate concentration, denitrifying bacteria, nitrite accumulation, acclimation, mineral carbon

## Abstract

Bacterial respiration of nitrate is a natural process of nitrate reduction, which has been industrialized to treat anthropic nitrate pollution. This process, also known as “microbial denitrification”, is widely documented from the fundamental and engineering points of view for the enhancement of the removal of nitrate in wastewater. For this purpose, experiments are generally conducted with heterotrophic microbial metabolism, neutral pH and moderate nitrate concentrations (<50 mM). The present review focuses on a different approach as it aims to understand the effects of hydrogenotrophy, alkaline pH and high nitrate concentration on microbial denitrification. Hydrogen has a high energy content but its low solubility, 0.74 mM (1 atm, 30 °C), in aqueous medium limits its bioavailability, putting it at a kinetic disadvantage compared to more soluble organic compounds. For most bacteria, the optimal pH varies between 7.5 and 9.5. Outside this range, denitrification is slowed down and nitrite (NO_2_^−^) accumulates. Some alkaliphilic bacteria are able to express denitrifying activity at pH levels close to 12 thanks to specific adaptation and resistance mechanisms detailed in this manuscript, and some bacterial populations support nitrate concentrations in the range of several hundred mM to 1 M. A high concentration of nitrate generally leads to an accumulation of nitrite. Nitrite accumulation can inhibit bacterial activity and may be a cause of cell death.

## 1. Introduction

Microbial denitrification is considered as more efficient at high nitrate concentration and more economical compared to physico-chemical techniques for nitrate removal (reverse osmosis, ion exchange, electrodialysis, chemical denitrification, adsorption methods) [1,2]. It is a respiration process leading to the reduction of nitrate while an electron donor (organic matter, hydrogen...) is oxidized. Nitrate is gradually reduced to nitrite, nitric oxide, nitrous oxide, and finally to dinitrogen in four successive reactions catalyzed by four microbial reductases. Microbial denitrification is still insufficiently investigated in non-conventional environments such as high nitrate concentrations, alkaline pH and hydrogenotrophic metabolism, despite its interest in the processing of various problematic industrial wastes. Therefore, the aim of this literature review is to give some indications of the possibilities of bacterial denitrification under these three conditions.

Several industries generate effluent and waste that can be highly concentrated in nitrate sometimes associated with non-advantageous environments such as alkaline pH and absence of organic matter. The disposal of radioactive waste deep underground faces a nitrate leaching issue in an alkaline environment. High nitrate concentrations (10 mM to 1 M) are expected in the vicinity of the radioactive waste, which could enhance radionuclide mobility [3,4,5]. The effluent from a stain-less steel plant was found to contain concentrations of up to 80 mM nitrate [6] and wastewater from the explosives industry can reach nitrate concentrations of up to 500 mM [7]. The management of such highly concentrated nitrate waste has become a major concern for these industries. High nitrate concentrations are fairly detrimental for microbial denitrification; the maximal nitrate concentrations tested in the literature range from hundreds of mM [8] to 1 M [9]. In particular, high nitrate concentrations cause nitrite accumulation, which is described as inhibiting, or even toxic, for bacteria [10,11,12]. Experimenting with high nitrate concentrations also requires high concentrations of the counter–ion Na^+^, K^+^, Ca^2+^, etc. to be added with nitrate. Caution is necessary here as there is no consensus on the effect of these cations [3].

Alkaline pH can be found in alkaline wastewaters [13], marsh soil management [14,15], alkaline lakes [16], and in disposal facilities for radioactive wastes. The alkaline environment strongly affects microbial denitrification. As the pH affects the functioning of all enzymes, it can also create an imbalance between the reduction kinetics of the four reductases of denitrification. Alkaline pH thus causes accumulation of metabolic intermediates such as nitrite. The maximal pH tolerated by bacteria, even alkaliphilic ones, is around pH 11.5 to 12 [17].

Hydrogen is an alternative energy source replacing organic matter for denitrifying bacteria. In subsurface environments or in industrial contexts such as a radioactive waste underground repository, organic matter concentrations can be low to negligible, while hydrogen can be generated through water radiolysis, mineral reactions, or iron corrosion [18,19,20,21]. Hydrogenotrophic denitrification has also aroused interest in the field of wastewater treatment as it results in water with low organic matter content [22,23]. Such denitrification (without organic matter) is considered to result in slower nitrate reduction and bacterial growth kinetics than heterotrophic denitrification (with organic matter) [24,25]. This is mainly due to the low hydrogen solubility [26,27] and the necessity for hydrogenotrophic bacteria to assimilate inorganic carbon for growth [28].

## 2. Definition and Biochemical Aspects of Denitrification

**Figure 1:**
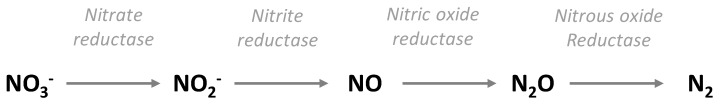
Overview of the four steps of microbial denitrification.

Microbial denitrification is a biological respiration process involving the successive reduction of nitrogen compounds: nitrate, nitrite, nitric oxide, and nitrous oxide, to nitrogen. The four steps are catalyzed by four different enzymes (reductases) (Figure 1) [29].

Microbial denitrification is generally carried out by heterotrophic bacteria using, for example, acetate as the electron donor (reaction (1)). When the environment is more restrictive, without organic matter, hydrogen becomes an alternative source of electrons for bacteria [23] (reaction (2)). Microbial denitrification, whether heterotrophic or hydrogenotrophic, is always accompanied by the production of OH^−^ ions, which affect the pH of the environment if it is not buffered.

5 CH_3_COOH + 8 NO_3_^−^ → 10 CO_2_ + 4 N_2_ + 6 H_2_O + **8 OH^−^** (1)

2 NO_3_^−^ + 5 H_2_ → 4 H_2_O + N_2_ + **2 OH^−^** (2)

**Figure 2:**
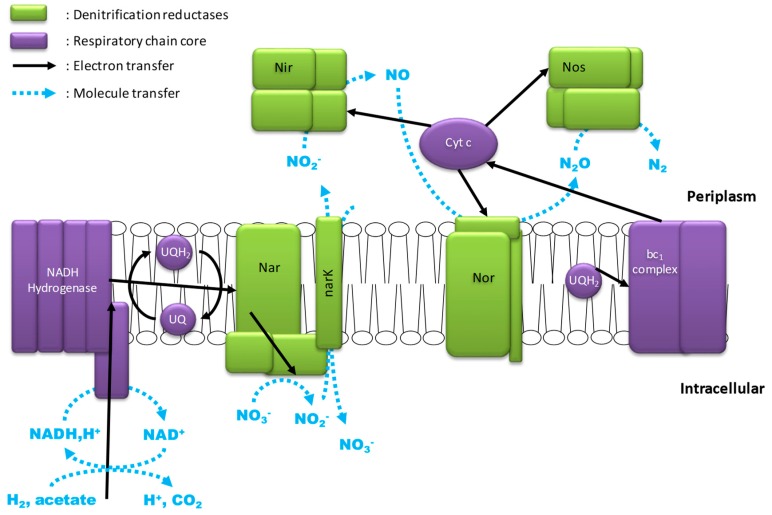
Schematic representation of the canonical respiratory chain of denitrification after [29,30,31].

Nitrate respiration is the process in which electrons are transferred from a donor (organic matter, hydrogen) to the nitrate acceptor (Figure 2). This redox reaction along the respiratory chain cogenerates a gradient of protons through the bacterial cell membrane, which is finally converted into energy in the form of ATP by ATP synthase [30].

Firstly, the electrons from the primary source of electrons (acetate, hydrogen, methanol, etc.) regenerate co-enzymes, such as NADH, H^+^. NADH, H^+^, or other potential electron donors such as succinate, which transfer their electrons to the respiratory chain [31]. Then electrons are carried through the respiratory chain by three types of electron transporters: (i) the Coenzyme Q known as Ubiquinone (UQ) in its oxidized state and Ubiquinol (UQH_2_) in its reduced state, (ii) the cytochrome bc_1_ complex, and (iii) the cytochrome c (Cyt. c) family of proteins containing a heme c [31,32]. Each of them can interact with several reductases [29,30].

The first reductase is the nitrate reductase (Nar). Three types of bacterial Nar complexes have been described. The membrane complex NarGHI is a molybdoenzyme, the active site of which faces the cytoplasm. This complex is usually adjacent to the narK membrane protein; it is an NO_3_^−^/NO_2_^−^ antiporter that absorbs NO_3_^−^ and excretes NO_2_^−^, Figure 2. The periplasmic reductase Nap reduces nitrate but cannot contribute to the proton gradient. The last nitrate reductase (Nas), is a periplasmic complex quite distinct from other reductase systems, as it is used in assimilatory nitrate reduction [33]. The nitrite produced by the nitrate reduction is then reduced by nitrite reductase (Nir). Two periplasmic types of enzymes have already been described: cd_1_-nitrite reductase with a heme-based active site and nitrite reductase with a copper-based active site [34]. The product of nitrite reduction, nitric oxide, is transformed by the membrane nitric oxide reductase (Nor) which is a member of the heme-copper oxidases family also capable, of reducing oxygen [35]. The last reduction is made by nitrous oxide reductase. It is a periplasmic enzyme that contains two Cu cores [29,30,36].

## 3. Influence of High Nitrate Concentration on Denitrification

### 3.1. Regulation of Denitrification, Nitrite Accumulation

**Figure 3:**
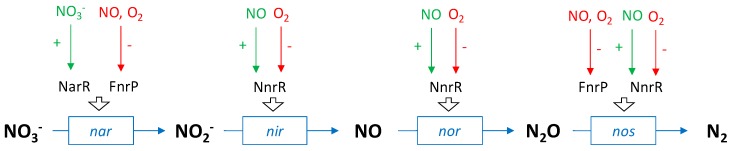
Schematic representation of the transcriptional regulation of the expression of genes encoding the different enzymes involved in denitrification in *P. denitrificans* [31,37].

Many denitrifying bacteria are facultative anaerobic: in the presence of oxygen the denitrification pathway is inhibited, and only aerobic respiration remains as it has the most efficient Gibbs free energy conservation. When O_2_ is low and NO_3_^−^ is available, denitrification is initiated. The denitrification intermediates NO_2_^−^ and NO are toxic compounds to bacterial cells [10,11,12,29], their internal concentration is regulated below cytotoxic levels to mM and nM respectively [30,31]. Consequently, O_2_, NO_3_^−^, NO_2_^−^ and NO are among the key signals that impact denitrification regulation. The regulation of the microbial denitrification at transcriptional level has been extensively studied using model denitrifying strains such as *Paraccocus denitrificans* or *Pseudomonas aeruginosa.* In denitrifying bacteria, the regulation of denitrification reductase gene transcription is managed by transcription factors of the FNR (Fumarate and Nitrate reductase Regulatory) family [37]. For example, in *P. denitrificans,* three types of FNR are involved: NarR (NO_3_^−^ and NO_2_^−^ sensitive), NnrR and FnrP (O_2_ and NO sensitive). Each one stimulates the transcription of different reductase genes (Figure 3).

In the well-studied denitrifying strain *P. aeruginosa*, the regulation is controlled by NarXL (NO_3_^−^ and NO_2_^−^ sensitive), ANR, DNR and NosR (NO and O_2_ sensitive) [38,39,40,41]. To sum up, in denitrifying bacteria, at the genomic level, there is a substrate regulation with NO_3_^−^ and a product regulation from NO and NO_2_^−^, the aim of which is to equilibrate the internal concentration of cytotoxic compounds such as NO_2_^−^ and NO [31]. Therefore, in culture under favourable conditions, intermediates as NO_2_^−^ and NO should not accumulate.

However, at a metabolic level, reductases competition can cause NO_2_^−^ or N_2_O accumulation. In the respiratory chain the transporters UQH_2_ can interact with three reductases (Nar, Nir, and Nos) and the transporters Cyt c with two reductases (Nir and Nos) [42,43]. UQH_2_ and Cyt c are therefore solicited by several electron acceptors at the same time and depending on the environmental conditions, such as the pH, electron transporters could transfer their electrons preferentially to one reductase rather than another [43,44]. Thus, at acidic pH (5.5), segmented denitrification was observed in *P. denitrificans* [43], i.e., the authors observed accumulation of NO_2_^−^ and N_2_O because UQH_2_ and Cyt c transferred electrons preferentially to some reductases, while at pH 8.5, there were no accumulations: UQH_2_ and Cyt c transferred electrons simultaneously to all reductases. Furthermore, the electron donor also impacts nitrite accumulation depending on the number of electrons it is likely to supply. In one study, nitrite accumulation occurred when a culture of *Pseudomonas stutzer*i was fed with 5 mM of acetate (two atoms of carbon) but did not occur with 5 mM of butyrate (four atoms of carbon) [45]. Butyrate is likely to release 20 electrons during its oxidation, while acetate releases only eight electrons, so it provides sufficient electrons and avoids competition among reductases for electron acceptance. Thus, accumulation of intermediates such as nitrite could occur because of environmental conditions and despite the strict regulation of transcription. To prevent nitrite accumulation within the cell, bacteria use transporters such as narK to excrete the nitrite [46].

In a mixed bacterial population, two distinct bacterial phenotypes can affect the nitrate and nitrite equilibrium: (i) *nitrate respiring* bacteria unable to reduce nitrite, (ii) *true denitrifying* bacteria reducing nitrate and nitrite to dinitrogen [47,48,49,50]. Growth rates are higher for *nitrate respiring* bacteria, and they rapidly become dominant. For example, growth rates three times higher have been observed for *nitrate respiring* bacteria [51]. Consequently, in the presence of nitrate, the domination of *nitrate respiring* bacteria causes nitrite accumulation. Once nitrate is consumed, the *true denitrifiers* continue to develop using nitrite and become dominant. In one study, activated sludge was acclimatized to nitrate concentrations of 190 mM at pH 7 to 9 in SBR reactors. The dominant bacteria in the inoculum were *nitrate respiring* bacteria while the bacteria remaining at the culture end were *true denitrifying* bacteria [47]. In this experiment, the accumulation of nitrite was pH-dependent; increasing with higher pH. Glass et al. hypothesized that the activity of *true denitrifiers* was slowed by alkaline pH. In summary, in a culture inoculated by a mixed population, the bacterial population and the culture conditions, such as alkaline pH, would impact nitrite accumulation. Therefore, in single strain or mixed population cultures exposed to high pH or high nitrate concentrations, the nitrite accumulations should not follow the same patterns.

Nitrite accumulation is problematic to denitrifying culture and in general for bacterial activity. The nitrite can inhibit and slow down bacterial activity at relatively low concentrations (tens of mM) [52]. It seems that nitrite can activate the synthesis of bacteriostatic molecules in *Bacillus cereus* [12]. Several other bacterial metabolic pathways such as nitrification [11,53,54] or methanogenesis [55] are also sensitive to the presence of nitrite. Concentrations of 10 mM to 100 mM of nitrite have been described as responsible for inhibitions of O_2_ assimilation, proline transport, or phosphorylating oxidation [10]. Moreover, nitrite can inhibit bacterial activity at a genomic level. Concentrations of 10 to 20 mM of nitrite caused a decrease in the concentration of mRNAs encoding for nitrification enzymes in the *Nitrosospira multiformis* and *Nitrosospira europaea* strains [11]. Other studies have reported nitrite toxicity [52] and even bacterial cell mortality in the presence of nitrite [10,56]. However, some bacteria could develop resistance to nitrite, for instance *P. denitrificans* tolerated 140 mM of nitrite in the presence of oxygen [57]. In conclusion, nitrite accumulation could be one of the major issues when denitrifying bacteria are cultivated with high nitrate concentrations.

### 3.2. High Nitrate Concentrations Reported in the Literature

In the literature, the maximum concentrations of nitrate tested in denitrifying cultures were of the order of hundreds of mM [45,48,51,58]. For example, an activated sludge culture was fed with an aqueous solution containing 645 mM of nitrate [59]. Lagoon samples (Oak Ridge, USA) initially concentrated at 645 mM of nitrate have been almost entirely denitrified (residual concentration of 0.8 mM) within a year [8]. Denariaz et al. [9] have reported the ability of a single strain culture of *Bacillus halodenitrificans* to survive at a concentration of 1.06 M of nitrate. In another study, *Rhodococcus sp.* was grown on 0.9 M of nitrate and 0.9 M of NaCl in aerobiosis [60]. These studies show that bacterial activity is possible at nitrate concentrations up to molar concentration in particular conditions. However, careful attention is needed as authors usually report nitrate concentrations from the feeding medium and not from the culture medium in contact with bacteria.

When using a synthetic medium for the cultivation of denitrifying bacteria or mixed consortia, the generation of such high concentrations of nitrate requires special attention to be paid to the counter-ion (NH_4_^+^, Na^+^, Ca^2+^, K^+^...) added with nitrate. There is no consensus on the effect of these highly concentrated cations on bacterial growth and activities. Francis and Hatcher compared the effect of three counter-ions (NH_4_^+^, Na^+^, Ca^2+^) on the denitrification kinetics of coastal sediments. Cultures underwent progressive nitrate increases up to 285 mM [3]. For each increase in the concentration of nitrate, denitrification kinetics were significantly higher when nitrate was added in the form of NH_4_NO_3_. Additions of NaNO_3_ and Ca(NO_3_)_2_ resulted in lower reduction kinetics. In addition, the salinity of the culture medium also has a significant effect on denitrifying bacterial cultures. In a culture with biomass initially adapted to 410 mM of NaCl, the NaCl concentration was increased from 8 to 1700 mM [61]. The results showed that the rate of denitrification decreased as the salt concentration increased. In contrast, some studies have reported rather high NaCl threshold concentrations: 1 M [62], 1.2 M [63], 1.9 M [64] and 4.25 M [9]. As an example, seawater contains 0.6 M of NaCl on average. Other studies observed denitrification enhancement by adding NaCl from 6.8 mM to 1.7 M [61] or calcium up to 3.75 mM [6].

Several studies have focused on the treatment of wastewater containing high nitrate concentrations from 100 to 1000 mM using activated sludge [6,59,63,64,65,66]. Experiments are presented in Table 1, showing the acclimation procedure and the nitrite build-up. The acclimation procedure made it possible to adapt bacteria to high nitrate concentrations by stepwise increases. As a result, in all experiments the nitrate was totally reduced, and the distribution of bacterial communities in activated sludge changed in favour of *nitrate respiring* bacteria [59].

**Figure 4:**
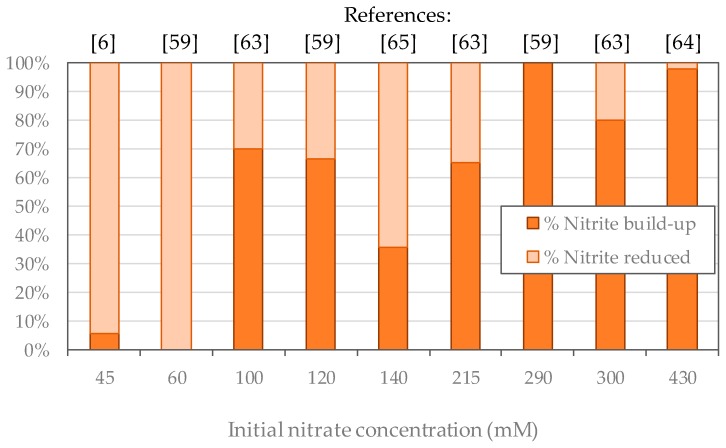
Proportions of nitrite reduced and accumulated according to the initial nitrate concentration in bacterial cultures after [6,59,63,64,65].

From Table 1 it is possible to estimate the proportion of reduced nitrite according to the initial nitrate concentration in the culture, as presented in Figure 4. Below 100 mM nitrate, the accumulation of nitrite does not exceed 90% of the initial nitrate concentration. Between 100 and 300 mM of nitrate, the accumulation of nitrite reaches between 40% and 70%. For concentrations greater than or equal to 300 mM, nitrite is no longer reduced except in one study [63]. Therefore, when experimenting on high nitrate concentration attention must be paid to the nitrite accumulation as it could reach concentrations as high as 420 mM. However, these studies focused on nitrate reduction, and longer experimental times might have allowed *true denitrifying* bacteria to proliferate and reduce nitrite.

## 4. Hydrogenotrophic Metabolism and Interactions with Denitrification

Hydrogen is an alternative energy source for denitrifying microorganisms in selective environments without organic electron donors. In order to survive in these environments, denitrifying bacteria must have the capacity to utilize nitrate as a nitrogen source, grow with inorganic carbon, utilize hydrogen as an electron donor and use nitrate as the terminal electron acceptor.

### 4.1. Hydrogen Oxidation Catalyzed by Hydrogenase Enzymes

**Figure 5:**
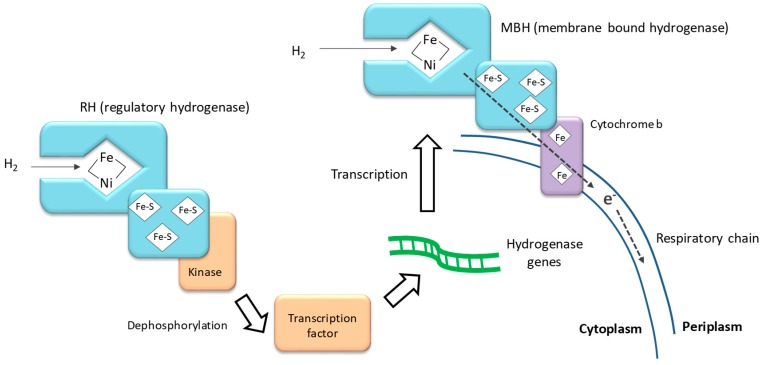
The regulatory [NiFe] hydrogenase and membrane-bound [NiFe] hydrogenase in *Ralstonia eutropha* after [69,71].

Hydrogen can be used as an electrons donor by different communities of bacteria reducing O_2_, NO_3_^−^, Fe^2+^, SO_4_^2−^, and CO_2_ as final electron acceptors [19,67]. Firstly, the reversible oxidation of hydrogen into protons (H_2_ ↔ 2 H^+^ + 2 e^−^) is catalyzed by bacterial hydrogenases. Then, the electrons are transferred to intermediates such as NAD^+^ or bc_1_ complex, which introduce the electrons into the denitrification respiratory chain. Hydrogenases consist of a protein part and a metal core constituting the active site of the enzyme. Hydrogenases are classified in three families differentiated by their metalcore, which is composed of one iron atom, alone or associated with one nickel or iron atom: [NiFe], [FeFe], [Fe]. The [NiFe] and [FeFe] hydrogenases have a similar domain organization, they are heterodimeric enzymes with an active site protected inside a large protein monomer and connected externally by a channel allowing only hydrogen to enter. The other, smaller monomer contains FeS clusters to transport electrons to the respiratory chain, Figure 5 [68,69,70,71]. The [Fe] hydrogenase contains a mononuclear metal center and is devoid of iron-sulfur clusters. It has only been identified in methanogenic Archaea [72]. The distribution of the three hydrogenase types in bacteria and archaebacteria are presented in Table 2. Hydrogen is rather uncommon in natural environments and is often associated with the absence of organic matter, high temperature, or high pressure, etc. Thus, the hydrogen source is utilized by a rather limited quantity of bacterial species. Most of the organisms studied as hydrogen-oxidizing denitrifiers belong to the phylum of Proteobacteria [23].

The regulation of hydrogenase production has been extensively studied in *Ralstonia eutropha* for industrial purposes [69,91,92]. In *R. eutropha* four [NiFe] hydrogenases have been identified: a membrane-bound hydrogenase (MBH) linked to the respiratory chain by a cytochrome b, a cytoplasmic soluble hydrogenase (SH), an actinobacterial-type hydrogenase (not well known yet) and a regulatory hydrogenase (RH) [87,91]. The RH forms a tight complex with a histidine protein kinase and acts as a hydrogen sensor. In the presence of hydrogen, the RH kinase complex enhances the production of MBH and SH by phosphorylation/dephosphorylation signals transmitted to MBH and SH transcription factors, Figure 5.

A similar regulation system is used by other bacteria, such as *Rhodopseudomonas palustris* [88]. However, many different regulatory pathways have been identified in bacteria. For instance, in *Cyanobacterium Synechocystis* three transcription factors regulate its hydrogenase: two positively acting regulators, LexA and AbrB1 and one repressor, AbrB2 [79]. In *Escherichia coli*, three transcription factors regulate its hydrogenase-1, ArcA and AppY enhance the hydrogenase production while IscR represses it. ArcA and AppY compete with IscR to bind with the hydrogenase gene promoter [93].

### 4.2. Mineral Carbon Assimilation

In environments devoid of organic substrates, hydrogenotrophic bacteria have to assimilate mineral carbon for growth. The enzymes involved in carbon assimilation are the carboxylases. The mineral carbon assimilation requires energy in the form of reduced co-enzyme (NADH,H^+^, FADH,H^+^) and ATP. For instance, in the Calvin cycle, 3 ATP and 3 NADH,H^+^ are consumed per equivalent of CO_2_ to produce glyceraldehyde-3-phosphate [94]. There are currently six known bacterial pathways leading to the assimilation of mineral carbon [49,50,95]:

- the reductive pentose phosphate (Calvin–Benson) cycle [94]
- the reductive acetyl-CoA (Wood–Ljungdahl) pathway
- the reductive citric acid cycle, the 3-hydroxypropionate bicycle
- the dicarboxylate/4-hydroxybutyrate cycle
- the 3-hydroxypropionate/ 4-hydroxybutyrate cycle.

Carboxylases are able to assimilate mineral carbon as CO_2_ or HCO_3_^−^. The availability of these forms is dependent on the pH, as CO_2_ can hydrate itself into carbonate species (H_2_CO_3_, HCO_3_^−^ and CO_3_^2−^) according to the pH. This hydration (H_2_O + CO_2_ ↔ H_2_CO_3_ ↔ HCO_3_^−^ + H^+^↔ CO_3_^−^ + 2 H^+^) causes acidification. Most bacterial carboxylases assimilate the CO_2_ form. However, at pH between 6.4 (pKa of (H_2_CO_3_/HCO_3_^−^)) and 10.3 (pKa of (HCO_3_^−^/CO_3_^2−^)), the HCO_3_^−^ form is dominant in solution. Bacteria have adapted by using carbonic anhydrases to catalyze the formation of CO_2_ from HCO_3_^−^ [96], or by using several carboxylases capable of fixing the HCO_3_^−^ form [97,98].

Mineral carbon can be supplied in aqueous solution either by bubbling CO_2_ (g) or by adding soluble carbonates (H_2_CO_3_, HCO_3_^−^, CO_3_^2−^). The continuous supply of CO_2_ (g) rapidly acidifies the pH of bacterial culture media [99]. In contrast, the addition of soluble carbonate buffers the solution. A study reported faster bacterial adaptation to hydrogenotrophy using HCO_3_^−^ as carbon source rather than CO_2_ (g) [100]. In addition, the mineral carbon supply must be balanced with a nitrogen supply in order to have an optimal carbon/nitrogen ratio for bacterial growth. In theory, 0.2 mg HCO_3_^−^-C/mg NO_3_^—^N would be required [23]. In practice, higher C/N mass ratios have been used in order to prevent carbon limitation [28]. Ratios should be chosen with care; C/N ratios that are too high can lead to alternative nitrate reduction pathways, such as nitrate reduction to ammonium, while C/N ratios that are too low lead to the inhibition of denitrification [23].

### 4.3. Comparison between hydrogenotrophic and heterotrophic denitrification

Interest in hydrogenotrophic denitrification for wastewater treatment has grown in recent decades, due to its low production of sludge [22,23,101]. In hydrogenotrophic cultures, denitrification and growth rate are lower than in heterotrophic cultures. In batch cultures of activated sludge, an initial concentration of 14 mM nitrate was reduced at 2.1 mM/d in heterotrophy and at 1.3 mM/d in hydrogenotrophy [25]. This difference can be explained by the additional energy expended for mineral carbon assimilation compared to organic carbon assimilation or the kinetic disadvantage of using hydrogen with low solubility. The aqueous solubility of hydrogen is 0.74 mM at 30 °C, thus hydrogen bioavailability can be limiting in fast biological processes [26]. In order to prevent hydrogen limitation, reactor designs have been optimized using porous membrane, hollow fiber, and silicone tube reactors [23]. These reactors are designed to improve the supply of hydrogen to bacterial cells. Thus, they result in better denitrification kinetics than simple batch reactors. In addition, based on Henry’s law, the utilization of high hydrogen pressure makes it possible to increase the hydrogen solubility. To illustrate this beneficial effect, using a continuous reactor fed with 1 mM nitrate, the nitrate reduction rates were respectively 43 and 170 mM/d at P_H2_ = 0.4 bar (1.5 bar total) and at P_H2_ = 1.3 bar (3 bar total) [102]. In conclusion, hydrogenotrophic bacterial cultures often result in lower denitrification kinetics than in heterotrophic cultures [22,24]. However, in some studies, the optimization of hydrogen transfer to bacterial cells has made it possible to obtain denitrification kinetics comparable to those of heterotrophic denitrification [102,103,104]. An overview of the nitrate reduction rate observed in the literature according to the pH and the nitrate concentration is presented in Table 3. Concentrations from 0.1 to 40 mM and pH from 6.5 to 9.5 have been explored. The maximal nitrate reduction rate did not exceed an order of magnitude of 100 mM/d, except for the experiment with high hydrogen pressure and very low nitrate concentration [102].

## 5. Influence of High pH on Denitrification

### 5.1. Basics of pH Effect on Denitrification

The pH impacts all enzymes that work properly at an optimal pH. In consequence, the majority of neutrophilic denitrifying bacteria have an optimal pH ranging between 7.5 and 9.5 [47,104,106,108]. At a lower pH, denitrification activity is slowed down. In the denitrification process, reductases or electron transporters are affected by the pH. Under the influence of the pH, electron transporters may preferentially give their electrons to specific reductases. For example, a study was carried out to measure nitrite reductase activity and nitrous oxide reductase activity according to pH variation from 6.4 to 9.2 [44]. Cytochrome c was more oxidized by nitrite reductase than by nitrous oxide reductase at pH < 7.3 and vice versa at pH > 7.3 [44]. Besides the acidic pH impact on the enzymes, the bacterial activity could be slowed by the formation of nitrous acid (HNO_2_) from nitrite. Nitrous acid is a cytotoxic compound that can easily cross bacterial membranes [57].

For pH values above the classical optimal pH range of 7.5 to 9.5, the denitrification kinetics recorded in the literature are generally slower and nitrite accumulations have often been observed [104,105]. In addition, the alkalinization of microbiological culture media is accompanied by an increase in the occurrence of precipitates. Denitrifying tests were performed for pH values from 7.7 to 9.5 [107]. At pH 9.5, in the presence of soluble carbonates, the increasing pH led to the precipitation of calcium carbonates, thereby modifying the bioavailability of carbonate and Ca^2+^ ions in the solution. Other precipitates (CaHPO_4_, Ca(H_2_PO_4_)_2_, Ca_3_(PO_4_)_2_, etc.) are likely to form depending on the pH and the nutrients that may be added [105]. All these precipitation phenomena could limit the nutrients available for proper bacterial denitrification.

On the other hand, bacterial denitrification has an effect on pH. The reactions below describe the reduction from nitrate to nitrite ((3) and (4)), then nitrite to nitric oxide ((5) and (6)), then nitric oxide to dinitrogen ((7) and (8)) with acetate or hydrogen. The nitrite reduction to nitric oxide ((5) and (6)) is the only reduction step that produces OH^−^ with either acetate or hydrogen [105]. Therefore, at alkaline pH, stopping the nitrite reduction would be a way for bacteria to avoid further pH increases. This would provide an explanation for the many observations of nitrite accumulations in denitrifying cultures performed in alkaline media.

4 H_2_ + 4 NO_3_^−^ → 4 NO_2_^−^ + 4 H_2_O (3)

CH_3_COOH + 4 NO_3_^−^ → 2 CO_2_ + 4 NO_2_^−^ + 2 H_2_O (4)

4 H_2_ + 8 NO_2_^−^ → 8 NO + **8 OH^−^** (5)

CH_3_COOH + 8 NO_2_^−^ + 2 H_2_O → 2 CO_2_ + 16 NO + **8 OH^−^** (6)

4 H_2_ + 4 NO → 2 N_2_ + 4 H_2_O (7)

CH_3_COOH + 4 NO → 2 CO_2_ + 2 N_2_ + 2 H_2_O (8)

However, in heterotrophic denitrification, organic matter (such as acetate) is oxidized to CO_2_, which has an acidifying action and could compensate for OH^−^ alkalinization. Therefore, calculations of pH were made to understand the pH evolution in heterotrophic cultures supplemented with acetate and a carbonate buffer. In the pH range of 8 to 14, considering that all the strong base OH^−^ reacts with CO_2_ to produce HCO_3_^−^ and then with HCO_3_^−^ to produce CO_3_^2−^, equation (1) can be rewritten by introducing equation (9).

5 CH_3_COOH + 8 NO_3_^−^ → 7 HCO_3_^−^ + 3 CO_3_^2−^ + 4 N_2_ + 4 H_2_O (9)

Therefore, the pH can be calculated from the Henderson-Hasselbalch equation by determining the final concentration of HCO_3_^−^ and CO_3_^2−^ depending on the nitrate reduced, Table 4.

Therefore, the pH can be expressed as in equation (10). If the nitrate concentration is significantly higher than the initial carbonate concentration, the pH tends to 10 (= 10.32 + log (3/7)). Therefore, during the denitrification with acetate, the pH may acidify or alkalinize depending on whether the initial pH is higher or lower than 10. This pattern is reported in the literature as “self-acidification” at alkaline pH [23,57,104] and “self-alkalinization” at acidic pH [58,109].

(10) $$\mathrm{pH}\;=10.32+\mathrm{Log}\left(\frac{{\left[{\mathrm{CO}}_{3}^{2-}\right]}_{i}+\frac{3}{8}\times \left[{\mathrm{NO}}_{3}^{-}\right]}{{\left[{\mathrm{HCO}}_{3}^{-}\right]}_{i}+\frac{7}{8}\times \left[{\mathrm{NO}}_{3}^{-}\right]}\right)$$

In the case of hydrogenotrophic denitrification, there is no CO_2_ production. The pH can only increase due to the production of OH^−^, which can react with HCO_3_^−^ to form CO_3_^2−^. Therefore, the pH from a hydrogenotrophic culture buffered with carbonate can be calculated from equation (2) and the Henderson-Hasselbalch equation, Table 5.

During hydrogenotrophic denitrification, as expressed in equation (11), the pH increases regardless of the initial pH. Moreover, the pH could also increase during mineral carbon assimilation. When hydrogenotrophic bacteria assimilate CO_2_ and HCO_3_^−^ for growth, the carbonate equilibrium is affected and the pH increases.

(11) $$\mathrm{pH}=10.32+\mathrm{Log}\left(\frac{{\left[{\mathrm{CO}}_{3}^{2-}\right]}_{i}+\left[{\mathrm{NO}}_{3}^{-}\right]}{{\left[{\mathrm{HCO}}_{3}^{-}\right]}_{i}-\left[{\mathrm{NO}}_{3}^{-}\right]}\right)$$

In conclusion, in hydrogenotrophic cultures, the pH would increase during the denitrification and during the mineral carbon assimilation. Consequently, hydrogenotrophic bacteria without any means of acidifying the pH are more sensitive to alkaline pH than heterotrophic bacteria, which are able to acidify pH above 10 in the presence of acetate.

An overview of the nitrate reduction rate observed in the literature in heterotrophic cultures is presented in Table 6. Concentrations from 10 to 1000 mM and pH from 5.5 to 12 are explored. The maximal nitrate reduction rate was an order of magnitude of 10 to 1000 mM/d. Higher ranges of pH and nitrate concentration were explored in heterotrophy than in hydrogenotrophy (see Table 3). The nitrate reduction rates, up to 5000 mM/d, were also higher in heterotrophy.

### 5.2. Bacterial Adaptations to Alkaline pH

**Figure 6:**
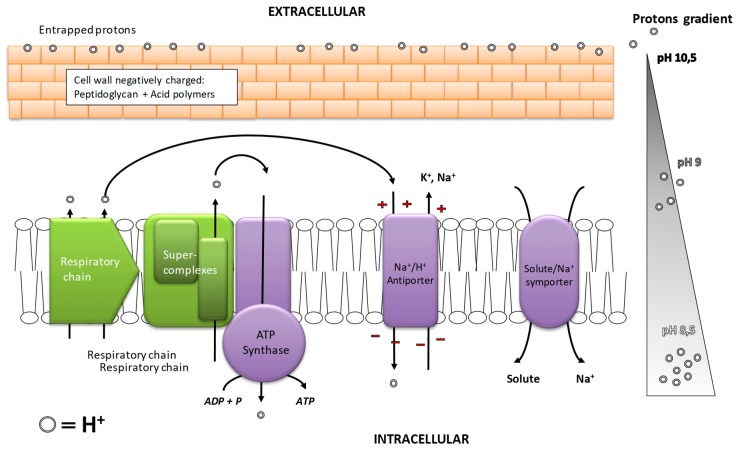
Protective mechanisms of *Bacillus sp.* cultivated at pH 10.5, adapted from after [111,117].

Most bacterial processes are significantly slowed down in an alkaline medium. However, some bacteria described as alkaliphilic grow optimally at pH above 9 [111]. Alkaliphilic bacteria such as *Bacillus sp.* were isolated from alkaline ecological niches [112] and have been extensively studied recently for industrial purposes [113,114]. Several protective mechanisms that allow alkaliphilic bacteria to maintain their metabolic activity at high pH can be illustrated by the example of the genus *Bacillus sp.* These mechanisms seek to keep the internal pH of the cells as low as possible and to adapt the internal enzymatic activity to high pH levels [115,116] (Figure 6).

In *Bacillus sp.* the first protective barrier, the bacterial wall, has additional acidic polymers and peptidoglycans installing a selective permeability to Na^+^ and H^+^ while blocking OH^−^ anions [115,116]. Then, on the bacterial membrane, transporters ensure that the pH in the cytoplasm is maintained as low as possible by a constant flow of protons inwards. In alkaliphilic bacteria, there are several types of membrane transporters allowing protons to be absorbed: Na/H anti-transporters, K/H anti-transporters, and the Mrp (Na/H) anti-transporter [117]. The Mrp anti-transporter is a super enzymatic complex encoded by several genes [117]. It has a crucial role in the absorption of protons and has been identified in several alkaliphilic bacterial strains [111,117]. These cellular mechanisms of protection would only generate a maximum difference of about 2 pH units between the cytoplasm and the culture medium [17,115]. Sturr et al. showed that the internal pH of *B. pseudofirmus* was maintained to 8.3 when the external pH was 10.8 [118]. However, this bacterium was no longer able to regulate its intracellular pH for external pH above 11.4.

Therefore, bacteria also need to adapt to the alkalinization of their internal pH to maintain their activity. A major challenge would be to adapt the respiratory chain, which at neutral pH generates a proton gradient used for ATP synthesis or molecule transports. However, at alkaline pH, the proton gradient is reversed. To maintain nutrient absorption, alkaliphilic bacteria use a gradient of Na^+^ ions. The sodium gradient created by membrane transporters replaces the proton gradient for the transport of nutrients (Figure 6). Thus, sodium is a key factor involved in the resistance to alkaline pH. Adaptations related to the functioning of ATP-synthase have also been reported to counter-balance the low concentration of protons in the periplasm [117]. In the respiratory chain, enzymes group together into super-complexes to facilitate proton transport to ATP-synthase and improve promiscuity with ATP-synthase. The cytoplasmic membrane is organized into micro-domains that allow the respiratory chain and ATP-synthase to be compartmentalized and fixed nearby. Finally, ATP-synthase, itself, is modified to improve its affinity for protons [117].

Amino acid substitutions were also observed on various enzymes not necessarily involved in respiration, allowing them to operate efficiently at alkaline pH [119]. The optimal pH for proteins of alkaliphilic bacteria is, therefore, higher than those of neutrophilic bacteria. Horikoshi [111] highlighted an extracellular protease with an optimal pH of 11.5. This enzyme was able to maintain its protease activity up to pH 13.0. Amino acids sequence analyses of a protease [120], an amylase [121] and a phosphoserine aminotransferase of *Bacillus sp*. showed a common trend in amino acid substitutions. Negatively charged amino acids tended to be replaced by neutral amino acids. Amino acids such as lysine were replaced by arginine, which has a higher pKa. At the protein structural level, an increase in the number of hydrogen or hydrophobic bonds was observed [122].

These adaptation strategies have mostly been demonstrated from specific alkaliphilic bacterial species of the genus *Bacillus*. These bacteria are not necessarily denitrifying. However, there are alkaliphilic denitrifying bacteria, such as *Halomonas desiderata*, which have been isolated from a soda lake [123]. This species has shown an ability to grow and catalyze nitrate reduction in aqueous media from pH 10 to pH 12 [124,125,126]. It is likely that this type of alkaliphilic denitrifying bacteria would use the same mechanisms as described for the genus *Bacillus*.

5.3. pH Threshold Values

Surprisingly, one of the main difficulties when experimenting with alkaline bacterial cultures is to create and maintain a high pH in the culture medium [17]. The use of buffers such as phosphate and carbonate can be an option based on their high pKa: pKa (HCO_3_^−^/CO_3_^2−^) = 10.32 and pKa (HPO_4_^2−^/PO_4_^3^) = 12.32. Besides the medium limitation, it seems that there is a limit between pH 11.5 and pH 12 beyond which the physiology of bacterial cells is no longer possible. Many alkaliphilic bacteria already have their cellular activity significantly slowed down above pH 11 [112,123,124]. For instance, several studies have compared bacterial reduction kinetics for pH values of 10, 11 and 12 [53,54]. A first study tested the reduction of Cr (IV) [53] with Lake Mono sediments (California, USA), a second tested the reduction of nitrate with Buxton sediments [54]. In both studies, the activity was slowed at pH 11 and no activity was observed at pH 12. In contrast, other works reported bacterial survival for pH 12 or even higher [114,124,127]. However, as stated by Sorokin, particular attention must be paid to the operating conditions and a critical eye kept on what is reported [17]: (i) the pH of the culture is sometimes not maintained during the experiment or even not indicated, (ii) the pH reported in the feed medium is likely to be different from the culture pH in contact with bacteria. For example, alkaliphilic bacteria isolated from an alkaline lake were cultivated at a pH adjusted to very high values (up to 13.2) [116]. But the pH decreased over time and bacterial growth was only observed when the pH had decreased below 12.0. In another experiment, the authors correctly indicated a difference of 2 pH units between the alkaline pH in the feeding medium and the pH in the culture [66].

## 6. Perspectives, Denitrification at Alkaline pH, with High Nitrate Concentration and with Hydrogen as Electron Source

**Figure 7:**
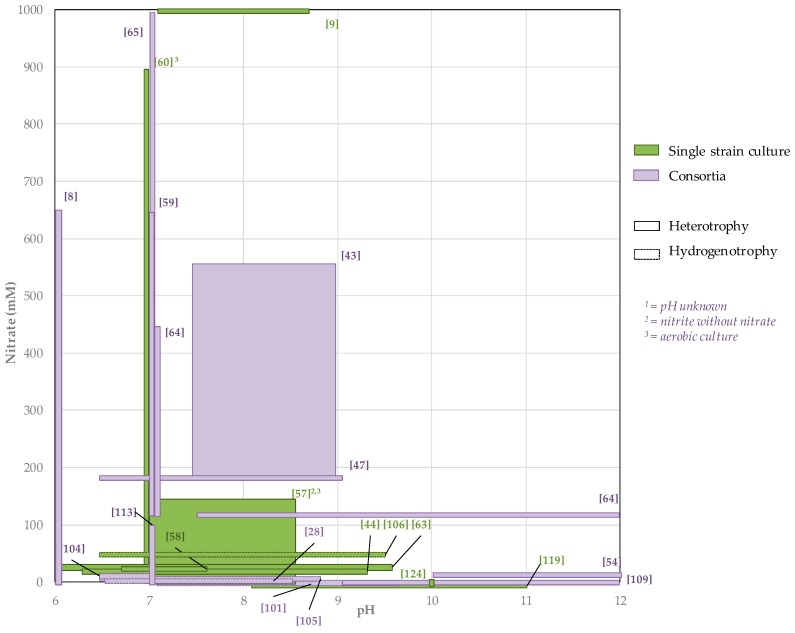
Literature overview of the experiments of bacterial denitrification conducted at alkaline pH and/or at high nitrate concentrations.

The impact of the association of high nitrate concentrations and alkaline pH on bacterial denitrification has not been well studied, especially with hydrogen as an energy source. Yet there is a strong interest in industrial perspectives, for nuclear waste repository management for instance [128,129]. In Figure 7, the different nitrate concentrations and pH investigated in the literature are summed up. It brings to light an important lack of knowledge concerning denitrifying cultures at high pH and high nitrate concentrations. In particular, there are no studies that have simultaneously tested a pH above 9 and a nitrate concentration above 120 mM. Therefore, future exploratory experiments conducted at high pH and high nitrate concentration should bring appreciable knowledge.

Despite the lack of experiments at high pH and high nitrate concentrations, speculations can be made about the behaviour of bacteria in this type of environment, based on the paragraphs above. Concentrations of the order of 100 mM and pH values above 9.5 are likely to cause nitrite accumulations (up to 100% of the initial nitrate concentration). Particular attention should be paid to monitoring nitrite as it is likely to inhibit bacterial activity at low concentrations (tens of mM). Moreover, while testing high nitrate concentrations, attention must be paid to the counter ion added with the nitrate as it could affect both pH and nitrate resistance. When testing alkaline pH, the pH must be buffered and must be closely monitored as denitrifying activity modifies the pH. Carbonates could be used as a buffer at alkaline pH, especially since they provide a mineral carbon source for bacteria in hydrogenotrophy. It seems unlikely that hydrogenotrophic denitrifying bacteria would be active at pH > 11, as the alkalinization concomitant with their activity would quickly raise the pH to the threshold around 11.5 to 12.

## 7. Conclusions

Exploring microbial denitrification in environments associated with alkaline pH, high nitrate concentration and hydrogen as electron donor seems possible at pH up to 11 and nitrate concentrations up to hundreds of mM. However, there is a lack of research associating these conditions and the potential negative effects of the three have generally been studied separately.

The maximal nitrate concentrations tested in the literature are generally about a few hundred mM of nitrate. At these concentrations, nitrite accumulation can reach 100% of the initial nitrate concentration. Nitrite is generally described as cytotoxic for bacterial cells at low concentrations (tens of mM). Some authors have succeeded in cultivating bacterial strains in culture media containing 1 M of nitrate. The counter ion added with nitrate, and more generally the salinity of the solution, is a major factor affecting the survival of bacteria. Some bacteria are tolerant to high salinities while others are inhibited by high concentrations of sodium. In addition, in a consortium, the composition of the bacterial population is decisive in the nitrite/nitrate balance.

In hydrogenotrophic cultures, denitrification and cell growth kinetics are generally slower than in heterotrophic cultures. One explanation is the availability of hydrogen, which is poorly soluble in aqueous media. The obligation for hydrogenotrophic bacteria to assimilate mineral carbon for growth is also a limiting factor. In addition, hydrogenotrophic denitrification appears to be more sensitive to high pH and more likely to cause nitrite accumulation than heterotrophic denitrification.

The pH is a determining factor for the survival of bacteria, the maximum limit values, below which microbial activity is possible, being between pH 11.5 and 12. The reduction of nitrite to nitric oxide generates alkalinity and, as a result, nitrite is likely to accumulate at alkaline pH. For most bacteria, denitrification is therefore incomplete for pH > 9.5, nitrite accumulation is observed, and reductions kinetics are slowed down. The whole denitrification process has an opposite impact on the pH if the electron donor is organic or hydrogen. Heterotrophic bacteria are able to acidify the pH when the initial pH is high, while hydrogenotrophic bacteria only alkalinize the pH. This crucial difference explains why hydrogenotrophic bacteria are more sensitive to alkaline pH.

## Figures and Tables

**Table 1:** Experiments with activated sludge cultures highly concentrated in nitrate, acclimation procedure and nitrite accumulation.

Experimental Protocol	Acclimation Procedure	Nitrate (In Culture)	Nitrite Build-Up	Ref.
Ca increase from 50 to 550 g/L at 45 mM nitrate, pH 8.5	Medium diluted x2 during 2 weeks	45 mM	max 2.5 mM	[6]
Nitrate increase to 580 mM and ionic strength from 0.8 to 3.0 in SBR, pH 9	Stepwise nitrate increase from 100 mM to 580 mM in about 6 weeks	100 mM 215 mM 300 mM	70 mM 140 mM 240 mM	[63]
Test at 140 mM nitrate in a batch reactor, pH 9/ Nitrate increase to 1000 mM in a continuous reactor	Stepwise nitrate increase Preculture: 14 mM to 140 mM in 5 weeks Culture: 140 to 1000 mM in 14 weeks	140 mM	50 mM ^1^	[65]
Nitrate increase to 640 mM in SBR	Step-wise nitrate increase in the medium from 120 mM to 640 mM in 8 weeks	430 mM 290 mM 120 mM 60 mM	420 mM 290 mM 60 mM 0 mM	[59]
NaCl stress decrease from 11 to 0 % at 430 mM, in continuous reactor	Stepwise nitrate increase from 140 mM to 430 in 3 weeks	430 mM (influent)	70–360 mM (effluent)	[64]
pH increase from pH 7.5 to 12 in SBR	Step-wise pH increase from 7.5 to 11.5 in 8 weeks	60 mM	30–55 mM (high pH)	[66]

^1^ Results of the batch culture.

**Table 2:** Phyla producing [NiFe], [FeFe] and [Fe] hydrogenase, with examples of strains.

Phylum	Specie, Genus	Hydrogenase	Ref.
Crenarchaeota (Archaea)	*Thermoproteus neutrophilus*	[NiFe]	[73]
Euryarchaeota (Archaea)	*Methanothermobacter marburgensis* *Thermococcus sp.*	[Fe], [NiFe]	[74] [75]
Actinobacteria	*Streptomyces avermitilis*	[NiFe]	[76]
Aquificae	*Aquifex aeolicus*	[NiFe]	[77]
Chloroflexi	*Thermomicrobium roseum*	[NiFe], [FeFe]	[78]
Cyanobacteria	*Synechocystis sp.*	[NiFe]	[79]
Firmicutes	*Clostridium sp.*	[NiFe],[FeFe]	[80]
Proteobacteria	*Paracoccus denitrificans* *Thauera sp.* *Hydrogenophaga sp.* *Pseudomonas stutzeri* *Escherichia coli* *Ralstonia eutropha* *Rhodopseudomonas palustris*	[NiFe], [FeFe]	[81,82] [83] [84,85] [50] [86] [69,87] [88]
Thermotogae	*Thermotoga maritima*	[FeFe]	[89]
Spirochaetes	*Treponema primitia*	[FeFe]	[90]

**Table 3:** Overview of nitrate maximal reduction rates in hydrogenotrophic cultures testing different pH and nitrate concentrations ranges.

Inoculum	Experimental Set-Up	pH	Nitrate mM	Nitrate Maximal Reduction Rate	Ref.
Activated sludge	Continuous reactor, heterotrophy or hydrogenotrophy	6.5–8.7	0.8–2.3	ND	[105]
Consortium	Pressured Batch reactor	7.1	0.07–0.7	356.4 mM/d	[102]
*Alcaligenes eutrophus*	Continuous and batch reactors	7.1–9	1.8–3.2	50.0 mM/d	[27]
*Paraccocus denitrificans*	Semi-batch reactors	6.5–9.5	40	8.4 mM/gDW/d ^1^	[106]
Activated sludge	Batch reactors	6.4–7	0.5–14.3	5.5 mM/d	[104]
Activated sludge	Batch and continuous reactors	ND	14	1.3 mM/d	[25]
Activated sludge	Continuous reactor	7–9.5	1	31 mM/d	[107]
Activated sludge	Sequencing batch reactors	7–9.5	1.4	27.4 mM/d	[101]

^1^ Expressed in terms of dry weight.

**Table 4:** Calculation of pH in a culture buffered with carbonate and fed with acetate according to the reduced nitrate concentration.

Equivalents	[HCO_3_^−^]_produced_ ⇔ 7/8 [NO_3_^−^]_reduced_ [CO_3_^2−^] _produced_ ⇔ 3/8 [NO_3_^−^]_reduced_
Final carbonate concentrations	[CO_3_^2−^]_final_ = [CO_3_^2−^]_initial_ + [CO_3_^2−^]_produced_ = [CO_3_^2−^]_initial_ + 3/8 [NO_3_^−^]_reduced_ [HCO_3_^−^]_final_ = [HCO_3_^−^]_initial_ + [HCO_3_^−^]_produced_ = [HCO_3_^−^]_initial_ + 7/8 [NO_3_^−^]_reduced_
Henderson-Hasselbalch equation	$\mathrm{pH}=10.32+\mathrm{Log}\left(\frac{{\left[{\mathrm{CO}}_{3}^{2-}\right]}_{\mathrm{final}}}{{\left[{\mathrm{HCO}}_{3}^{-}\right]}_{\mathrm{final}}}\right)$
Final equation	$\mathrm{pH}=10.32+\mathrm{Log}\left(\frac{{\left[{\mathrm{CO}}_{3}^{2-}\right]}_{\mathrm{initial}}+\frac{3}{8}{\left[{\mathrm{NO}}_{3}^{-}\right]}_{\mathrm{reduced}}}{{\left[{\mathrm{HCO}}_{3}^{-}\right]}_{\mathrm{initial}}+\frac{7}{8}{\left[{\mathrm{NO}}_{3}^{-}\right]}_{\mathrm{reduced}}}\right)$

**Table 5:** Calculation of pH in culture buffered with carbonate and fed with hydrogen according to the reduced nitrate concentration.

Equivalents	[HCO_3_^−^]_consumed_ ⇔ [OH^−^]_produced_ ⇔ [NO_3_^−^]_reduced_ [CO_3_^2−^]_produced_ ⇔ [OH^−^]_produced_ ⇔ [NO_3_^−^]_reduced_
Final carbonate concentrations	[CO_3_^2−^]_final_= [CO_3_^2−^]_initial_ + [CO_3_^2−^]_produced_ = [CO_3_^2−^]_initial_ + [NO_3_^−^]_reduced_ [HCO_3_^−^]_final_= [HCO_3_^−^]_initial_ – [HCO_3_^−^]_consumed_ = [HCO_3_^−^]_initial_ – [NO_3_^−^]_reduced_
Henderson-Hasselbalch equation	$\mathrm{pH}=10.32+\mathrm{Log}\left(\frac{{\left[{\mathrm{CO}}_{3}^{2-}\right]}_{\mathrm{final}}}{{\left[{\mathrm{HCO}}_{3}^{-}\right]}_{\mathrm{final}}}\right)$
Final equation^1^	$\mathrm{pH}=10.32+\mathrm{Log}\left(\frac{{\left[{\mathrm{CO}}_{3}^{2-}\right]}_{\mathrm{initial}}+{\left[{\mathrm{NO}}_{3}^{-}\right]}_{\mathrm{reduced}}}{{\left[{\mathrm{HCO}}_{3}^{-}\right]}_{\mathrm{initial}}–{\left[{\mathrm{NO}}_{3}^{-}\right]}_{\mathrm{reduced}}}\right)$

^1^: does not apply if [HCO_3_^−^]_initial_ < [NO_3_^−^]_reduced_, in this case, the pH is directly calculated from [OH^−^].

**Table 6:** Overview of nitrate maximal reduction rates in heterotrophic cultures testing different pH and nitrate concentrations ranges.

Inoculum	Experimental Set-Up	pH	Nitrate mM	Nitrate Maximal Reduction Rate	Ref.
*P. denitrificans*	Batch reactor	ND	17	36 mM/d	[110]
*P. denitrificans*	Batch reactor, an/aerobic transition	5.5–9.5	25	60 mM/d	[43]
*P. denitrificans*	Batch reactor, high cell density	6.4–9.2	25	4887 mM/d	[44]
*P. denitrificans*	Continuous reactor an/aerobic transition	6.8–7.5	25	6 mM/d	[58]
Activated sludge	Sequencing batch reactors	6.5–9	192	600 mM/d	[47]
Activated sludge	Batch reactor	10–12	15	2 mM/d	[54]
Activated sludge	Sequencing batch reactors	7.2	120–645	1710 mM/d	[59]
Activated sludge	Sequencing batch reactors	7.5–12	120	1177 mM/d	[66]
Activated sludge	Sequencing batch reactors	7.5–9	192–580	564 mM/d	[63]
*Bacillus halodenitrificans*	Batch reactor	7.5–9	1006	ND	[9]
Activated sludge	Sequencing batch reactors	8.5	42	137 mM/d	[6]
Activated sludge	Expanded granular sludge bed	6–8	142–1000	99.9 % removal efficiency	[65]

## Abbreviations

FNR	Fumarate and Nitrate reductase Regulatory (also NarR, NnrR and FnrP)
UQ	Ubiquinone
NAD^+^	Nicotinamide adenine dinucleotide
FAD^+^	Flavin adenine dinucleotide
ATP	Adenosine triphosphate
Cyt. c	Cytochrome c
Nar	Nitrate reductase (NarGHI, NapAB and NasA are also different nitrate reductases)
Nir	Nitrite reductase (NirS and NiK are different nitrite reductases)
Nor	Nitric oxide reductase
Nos	Nitrous oxide reductase
